# Health Exhibition at Leeds

**Published:** 1923-12

**Authors:** 


### HEALTH EXHIBITION AT LEEDS.

A Health Exhibition has been held at the Drill Hall, Leeds, and has attracted much attention. The Public Health Department was especially instructive and included a model hygienic shop counter, fly-proof and ice-cooled, the model cow shed, a smoke-recording apparatus, a demonstration of food values, and a dozen other features conveying lessons to the eye at once. Personal cleanliness, as assured at the disinfecting stations, was illustrated by a very nice model, and the evolution of modern housing shown by models to scale of an area with houses crammed seventy to the acre, and of the post-war housing estates with ten houses to the acre, permitting air and sunlight about each house. A model ice-cream factory and a model dairy were also shown. A splendid model of a ward at the Killingbeck Sanatorium was made by Mr. Geo. Jackson, and fully equipped with 36 tiny cots and all fittings by the staff.

